# Gait Pattern Analysis: Integration of a Highly Sensitive Flexible Pressure Sensor on a Wireless Instrumented Insole

**DOI:** 10.3390/s24092944

**Published:** 2024-05-06

**Authors:** Partha Sarati Das, Daniella Skaf, Lina Rose, Fatemeh Motaghedi, Tricia Breen Carmichael, Simon Rondeau-Gagné, Mohammed Jalal Ahamed

**Affiliations:** 1 Mechanical, Automotive & Materials Engineering, University of Windsor, Windsor, ON N9B 3P4, Canada; psdas@hollandcollege.com (P.S.D.);; 2 Department of Chemistry and Biochemistry, University of Windsor, Windsor, ON N9B 3P4, Canada

**Keywords:** gait sensing, capacitive sensor, insole, gait phase monitoring, Ag electrode, micropatterned PDMS, wireless

## Abstract

Gait phase monitoring wearable sensors play a crucial role in assessing both health and athletic performance, offering valuable insights into an individual’s gait pattern. In this study, we introduced a simple and cost-effective capacitive gait sensor manufacturing approach, utilizing a micropatterned polydimethylsiloxane dielectric layer placed between screen-printed silver electrodes. The sensor demonstrated inherent stretchability and durability, even when the electrode was bent at a 45-degree angle, it maintained an electrode resistance of approximately 3 Ω. This feature is particularly advantageous for gait monitoring applications. Furthermore, the fabricated flexible capacitive pressure sensor exhibited higher sensitivity and linearity at both low and high pressure and displayed very good stability. Notably, the sensors demonstrated rapid response and recovery times for both under low and high pressure. To further explore the capabilities of these new sensors, they were successfully tested as insole-type pressure sensors for real-time gait signal monitoring. The sensors displayed a well-balanced combination of sensitivity and response time, making them well-suited for gait analysis. Beyond gait analysis, the proposed sensor holds the potential for a wide range of applications within biomedical, sports, and commercial systems where soft and conformable sensors are preferred.

## 1. Introduction

Analyzing gait phases is a technique used in biomechanics, clinical evaluation, rehabilitation, sports science, and research. Gait phase analysis provides valuable perspectives on human movement, assisting in diagnosing conditions, planning treatments, designing assistive devices, and enhancing overall mobility and quality of life. These valuable data serve multiple purposes, such as tailoring appropriate therapies for patients and enhancing gait stability in sports, patients, and everyday activities [1,2]. The study of human gaits encompasses an in-depth analysis of human movement, including the identification, description, and evaluation of the specific characteristics of human locomotion including walking patterns, the measurement of kinematic aspects across various phases of walking, and the assessment of musculoskeletal functions. Consequently, gait analysis is instrumental in biomedical engineering for the investigation of human locomotion. This wide-ranging utility has sparked significant interest among researchers and clinicians alike, to find sensors and instrumentations for gait analysis [3,4,5,6,7]. V. Bucinskas et al. devised and constructed a pressure sensor for assessing human gait by examining foot pressure distribution. This sensor allows for the evaluation of key gait parameters such as step rhythm, step size, weight distribution between heel and forefoot, and timing of gait phases. Gait patterns can be identified by analyzing sensor data in the time domain and extracting key parameters like pulse amplitude, pulse width, slope of the pulse front edge, and temporal shifts between pulses.

Currently, different wearable and non-wearable sensors are used to monitor and analyze gait patterns. The acceleration of the human body produced during gait can be measured using wearable sensors, such as an inertial measurement unit (IMU) and some other types of force-sensitive resistor (FSR) [8,9]. While being efficient, most of the previously designed sensors have limitations in terms of sensitivity, accuracy, dynamic range, or durability, thus affecting the overall performance of the sensor and suitability for gait analysis [10]. For example, Tao et al. described a smart insole and its microfabrication process to produce a sensor array to monitor the gait [11].

However, these sensor arrays have limitations in terms of resolution, reproducibility, or scalability, which can also affect the reliability and cost-effectiveness of the sensor array. To solve the problem of the above sensors, numerous studies have been performed on the creation of a capacitive pressure sensor (CPS) for gait monitoring. CPS works by sensing the variation in capacitance caused by the movement of the distance of two parallel conductive plates. The corresponding force is then calculated directly from the measured capacitance.

Utilizing CPS as wearable sensors offers numerous advantages, such as extending sensor lifespan and reducing production costs by introducing cost-effective rapid manufacturing processes [12,13,14,15,16,17,18,19]. Additionally, CPS exhibits superior sensitivity and lower linear error rates compared to FSR. Jia et al. reported a flexible CPS using 3D printing technology on paper for foot pressure monitoring [12]. Although this work achieved promising outcomes, it did not characterize walking patterns, preventing the comprehensive monitoring of the gait signal. In a previously reported sensor system from Samarentsis et al., a 3D-printed CPS-based smart insole was used, and displayed rapid response and relaxation times, as well as adaptability to various gait speeds [13]. In their study, the authors integrated sixteen pressure sensors hooked on a 3D-printed design to create a smart insole that effectively mapped the dynamic plantar pressure and could differentiate between different gait phases. However, the 3D-printed sensor has limited flexibility, and a used a thicker sole that can affect user comfort and wearability. As highlighted in these works, gait sensors can often face limitations related to stability, durability, nonlinearity, and hysteresis [20,21,22,23,24].

To tackle these challenges, and to increase the conformal fitting and sensor sensitivity, there has been a growing interest in flexible pressure sensors [25,26,27,28,29,30,31,32,33,34,35,36,37,38]. Zeng et al. devised a flexible capacitive pressure sensor characterized by its uncomplicated structure, economical production, broad operational scope, exceptional repeatability, and heightened sensitivity. This was achieved through a simple method for preparing the dielectric layer and the use of inexpensive chemical materials. Their findings demonstrate that the sensor exhibits robust responsiveness to both static and dynamic pressures, with repeatability. Notably, the sensor showcases exceptional dynamic response (80 milliseconds) and recovery (55 milliseconds). Within the pressure range of 0–10 kPa, the sensor demonstrates a sensitivity of up to 0.3199 kPa^−1^, and even within the range of 15–65 kPa, it maintains a sensitivity of 0.0779 kPa^−1^. Among the flexible polymer materials used in such flexible pressure sensors, polydimethylsiloxane (PDMS) is widely employed due to its stability, elasticity, dielectric constant, and recovery properties. Due to these key properties, several research efforts have focused on the development of embedded sensors for measuring, monitoring, diagnosing, and analyzing gait in rehabilitation settings. For instance, Park et al. fabricated a resistive-based foot pressure sensor that uses a highly sensitive strain sensor embedded in an elastomer material, along with a 3D-printed frame and stainless steel [39]. The sensor is composed of a flexible polymer substrate with a thin metal layer deposited on top and then layered with insulating material. The device can function as a real-time monitoring system by integrating a pressure visualization program and offers the flexibility to customize foot pressure measurements up to 160 kPa across five or more areas on the sole. Researchers have also proposed different material-based piezo-resistive and resistive sensors, multilayer graphene, and MWCNT/PDMS nanocomposite sensors to quantify gait pressure mapping data while walking and standing [40,41,42,43]. Overall, these studies demonstrate that the gait pressure dissemination is higher within the heel and forefoot when standing, then shifts to the whole foot and finally to the forefoot during the first occurrence of the gait cycle. Jung et al. proposed a foot pressure measurement method using printed flexible piezoresistive sensors [44]. In that work, the sensing parts of the gait insole were created through a carbon-based composite. Other types of sensors with multilayered construction have been published for gait monitoring [45,46]. Notably, because of the multilayer fabrication processes often required, these sensors often require a thicker insole and can be challenging in terms of flexibility and conformability. Our attention was on creating soft and flexible sensors that can be made inexpensively using methods like printing or molding, which makes them affordable for mass production. These sensors, if developed, could bring new ideas and advancements to areas like healthcare, robotics, wearable tech, automotive, and more [47,48]. They could be used in various devices and systems, even those with complicated shapes or parts that move, making them useful across different industries. Hence, despite numerous recent advancements in the design and production of flexible capacitive sensors, the design and fabrication of these sensors in a simple manufacturing process that also delivers device flexibility and adequate sensitivity remains a significant challenge.

**Figure 1:**
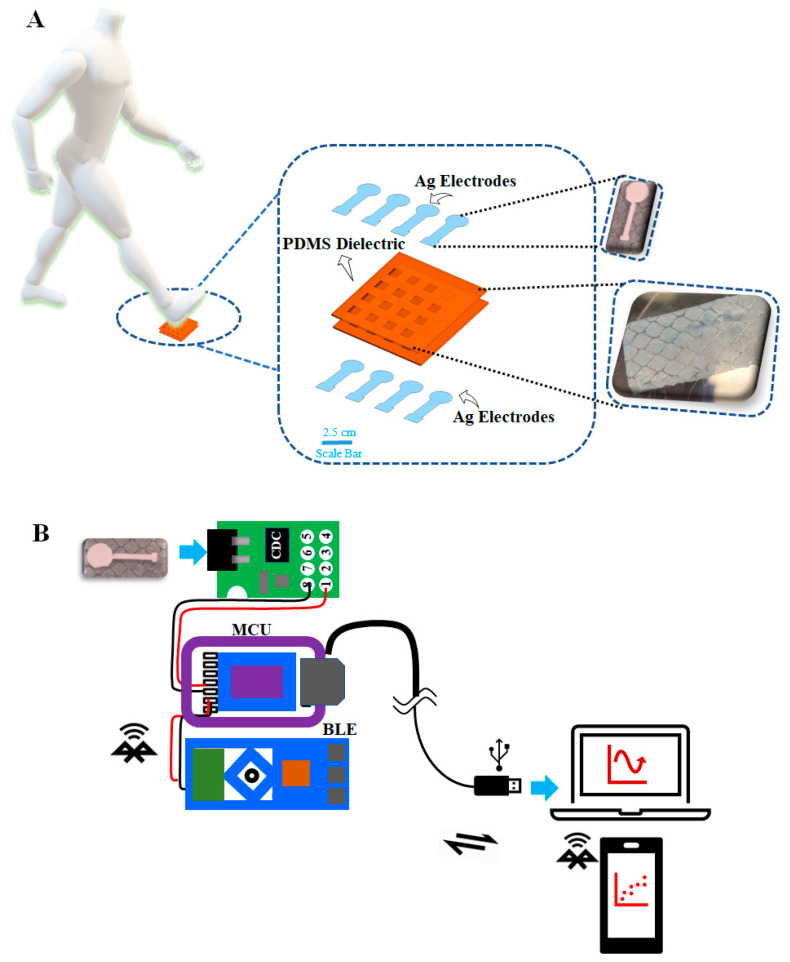
(**A**) Graphic demonstration of the wearable gait monitoring system, the inset shows the image of the electrode and the micropatterned PDMS dielectric layer. (**B**) Front-end electronics for the wearable gait monitoring system with wired and wireless options.

Herein, we report a high-performing, soft, and wearable capacitive pressure sensor made of PDMS molding combined with screen-printed electrodes through a basic PDMS mold transfer microfabrication method. A microstructured PDMS layer is utilized as the flexible dielectric material, while the patterned electrodes are obtained via screen-printing. Additionally, this pressure sensor includes wireless capability. The flexible capacitive pressure sensor exhibits high sensitivity and linearity under low pressure (SS_1_ = 0.06 kPa^−1^, R^2^ = 0.96) and high pressure (SS_2_ = 0.006 kPa^−1^, R^2^ = 0.92) and good stability. The sensor’s response and relaxation durations were very short under low and high pressure (200 ms and 175 ms, 200 ms and 100 ms, respectively). In addition to the fabrication of the device, our work also investigates the development of the pressure sensor to be used as an insole for gait analysis. Figure 1 describes the diagram of our smart insole system.

The wearable gait monitoring system includes the PDMS micropatterned dielectric layer-based soft capacitive sensor, which can measure both stationary and dynamic gait phases. A data acquisition system is also included, which can read data from four sensors, transmit the data wirelessly using Bluetooth Low Energy (BLE), and display the data on a mobile Android application (Figure 1B). To assess the system’s performance, a range of static and dynamic movements, such as different postures, walking, turning, falling, and going upstairs, were examined. Our findings demonstrate that the system is capable of continuously monitoring and distinguishing various plantar pressure distributions. The flexible insole, based on the typical arch foot architecture, incorporates four sensors located at the heel, lateral rear foot, midfoot, and front foot. The foot pressure over the pressure sensor was examined by detecting the change in capacitance of the pressure sensors when pressure was applied, revealing that the forefoot and heel experience greater pressure when a person stands in a normal stance. Gait phases and patterns were identified based on capacitive responses to static and dynamic plantar pressures from the instrumented insole. This work opens new applications in fields that include wearable medicine, detection of sports injuries, athlete training, design of sports equipment, and other related areas.

## 2. Materials and Methods

### 2.1. Sensor Fabrication

**Figure 2:**
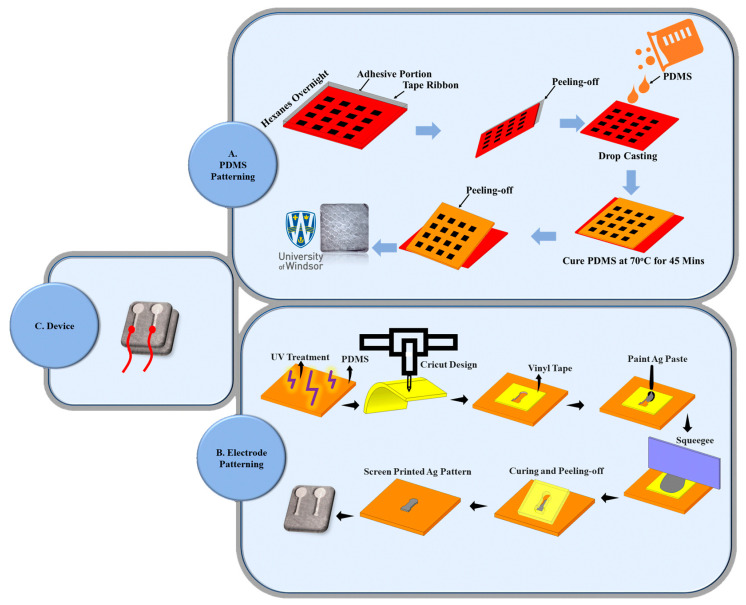
Fabrication of the capacitive pressure sensor. (**A**) The fabrication process of micropatterned PDMS. (**B**) The fabrication process of screen-printed electrode patterning. (**C**) The fabricated device.

The proposed sensor is fabricated through the application of micropatterned PDMS and screen-printed silver (Ag) paste on PDMS, as shown in Figure 2. The proposed device, along with the electronics, is designed for continuous and precise gait tracking in everyday life. Figure 1 illustrates the process wherein signals from the foot are obtained, verified, wirelessly transmitted, and subsequently processed to extract gait characteristics for health monitoring and offline analysis.

To create the micropatterned dielectric layer in our sensor, we use a soft molding technique with PDMS and a tape mold [49]. This process entails pouring liquid PDMS into the pattern-containing mold, followed by baking and cross-linking the material. Figure 2A details the steps for creating the micropatterned PDMS. Initially, the adhesive part of the tape is removed, and the tape ribbon (Scotch Tape) is dissolved in hexanes overnight. Then, a blend of PDMS elastomer, specifically Sylgard^®^ 184 from Dow Corning (Midland, MI, USA), composed of a base and curing agent, is poured onto the tape ribbon. After degassing in a vacuum desiccator, the mixture is cured at 70 °C for 45 min. This procedure yields a PDMS film that can be readily peeled off from the tape ribbon, resulting in the formation of the inverse tape structure. In this project, we augmented the area and thickness of the micropatterned dielectric layer to ensure a larger surface area, which is conducive to measuring a wide range of pressures.

The top and bottom electrodes of our capacitive sensor were fabricated using a screen-printing technique. Figure 2B describes the fabrication process of the screen-printed Ag electrode. The silver electrodes were fabricated using doctor blade screen printing. We designed the preliminary pattern using AutoCAD 2022 software and replicated it in Cricut Design Space software [50]. Subsequently, a Cricut machine was utilized to create the vinyl shadow mask essential for screen printing. Prior to the printing process, the micro-patterned PDMS underwent a 10 min UV/ozone treatment on PDMS film. Ag paste was then generously applied to the top of the vinyl shadow mask affixed to the PDMS, followed by the removal of excess paste using a squeegee. Upon peeling off the vinyl mask, the desired electrode pattern was revealed. Finally, the printed electrodes underwent curing at room temperature for 20 min, resulting in the completion of the final fabricated device, as illustrated in Figure 2C.

### 2.2. Working Principle

We developed a pressure sensor based on the capacitive principle, employing two PDMS electrodes with Ag patterns applied via screen printing. These electrodes are separated by an air gap and PDMS material. The operating principle of this capacitive pressure sensor is simple: when the distance between the Ag-patterned electrodes changes, the capacitance also varies (see Figure 1). This relationship is described by the formula C = ∈A/D, where C represents capacitance, ε stands for the relative dielectric constant of the material, A denotes the electrode area, and D signifies the distance between the electrodes. When pressure is applied to the electrodes, temporarily reducing the gap between them, if the material between the electrodes is elastically compressible, this change triggers a corresponding alteration in capacitance. Furthermore, the change in capacitance directly corresponds to the variation in distance and, thus, to the pressure change. By tracking these capacitance fluctuations, we can precisely measure pressure alterations, enabling the development of pressure-sensing devices.

### 2.3. Electrical and Mechanical Characterization

The electrical and mechanical characteristics of the sensor were analyzed using various device characterization approaches. This included assessing linearity, conductivity, and flexibility in both low- and high-pressure regions. Multiple sensors were fabricated and evaluated to select the most suitable one based on performance criteria. Characterization involved viewing the internal structure using a scanning electron microscope and measuring capacitance changes under applied pressure using a force gauge and an LCR meter.

Achieving optimal performance, ensuring reliability, adapting to diverse environments, and meeting specific application requirements all rely on the fundamental electrical and mechanical assessment of flexible pressure sensors. This characterization process is crucial in driving the progress of sensor technologies, enhancing their effectiveness and reliability. By altering the mechanical and electrical parameters, the proposed pressure sensor with a linear response and high sensitivity can be obtained. Figure S1 shows the characterization and measurement setup. The thickness of the dielectric layers is approx. 3 mm, and the size of the sensor is approx. 2.5 cm × 4 cm. In addition, the outer diameter of the sensor and the diameter of the detection area (i.e., the electrodes) were approximately 2.5 mm and 7 mm, respectively. The pressure sensor is made by connecting wires to two electrodes using conductive copper tape. The performance assessment of the pressure sensor fabricated in this study focused on three key parameters: linearity, conductivity, and flexibility. The sensor was characterized in both the low-pressure and high-pressure regions. In the low-pressure range, sensitivity was determined by applying forces ranging from 0 kPa to 25 kPa on top of the device. In the high-pressure range, sensitivity was evaluated by applying a force ranging from 30 kPa to 360 kPa. Numerous sensors were fabricated, incorporating different combinations of dielectric layers and electrode materials. Based on the performance evaluation of each pressure sensor, the most suitable one was selected. We used a field emission scanning electron microscope (S4800, Hitachi, Japan) to view the internal structure of the sensors. To assess and measure the flexible pressure sensor, a combination of a moving stage (Mark-10 ESM303) and a force gauge (M2, Force gauge, 500 kPa) was employed, as depicted in Figure S1. On both sides, the screen-printed film was covered with silver paste and tied to copper wires in order to measure capacitance. We recorded the capacitance changes in the sensors connected to the LCR meter (E4980A Precision LCR Meter) under 1 V and 1 kHz. We used the force gauge through a moving stage to apply pressure, while the LCR meter monitored the real-time capacitance characteristics of the fabricated sensor. For data acquisition from the sensors, we opted for the commercially available Arduino Nano board, a compact and small board based on the ATmega328 microcontroller.

### 2.4. Hardware and Wireless Data Transmission

The experimental setup included an insole integrated with the sensor device and a 3D-printed box housing the PCB and system components. This setup enabled optimal performance and portability, essential for real-world applications. To facilitate wireless data transmission, we employed Bluetooth Low Energy (BLE), a standardized protocol operating at 2.4 GHz. The complete hardware was enclosed in a 3D-printed box. We utilized DesignSpark Mechanical 2022 software to create a three-dimensional enclosure, specifically a box, to house the printed circuit board (PCB) and system components. The overall setup consisted of an insole and a 3D printed box that accommodated the PCB. This choice proved beneficial for ensuring optimal performance within a confined space, particularly when enclosed in a plastic box. To enhance portability, we designed the data acquisition device to be rechargeable and portable, utilizing lithium polymer (Li-Po) batteries that included built-in protection circuitry.

In summary, the fabrication process involved creating micropatterned PDMS and screen-printed Ag electrodes, while the sensor principle relied on capacitance changes to measure pressure variations. Electrical and mechanical characterization ensured performance optimization, while hardware and wireless data transmission facilitated practical applications in gait tracking and health monitoring.

## 3. Results and Discussion

### 3.1. Electrical and Mechanical Properties

The electrical characterization of the screen-printed electrode is shown in Figure S2. The resistance of silver (Ag) patterned electrodes changes when bent on PDMS. The bending angle was from 0 to 75 degrees, where it exhibits 3Ω at 45 degrees. It is demonstrated that even though there was a large deformation of the electrode, the resistance was low and stable.

### 3.2. Sensor Performance and Characteristics

Precise, reliable, and meaningful gait measurements in both high and low-pressure regions, essential for detecting human movement, require soft and flexible sensors with high sensitivity and linearity [13,14]. These characteristics ensure the sensor’s efficacy in accurately capturing the nuances of human motion, offering valuable insights applicable to both clinical and research settings. The proposed sensor with micropatterned PDMS showed high sensitivity and linearity. Due to the micropatterned structure of the PDMS, the separation between the two electrodes could easily be changed, resulting in good sensitivity through capacitance change. A thinner and more flexible sensor is preferred for a conformal fit inside the shoe and for better sensitivity. We reduced the thickness of a pressure sensor by using thin and flexible polymers (PDMS) and thin patterned electrodes. To enhance durability, we enhanced the wearability of the sensors by embedding them within a protective casing.

### 3.3. Sensitivity and Linearity Evaluation

**Figure 3:**
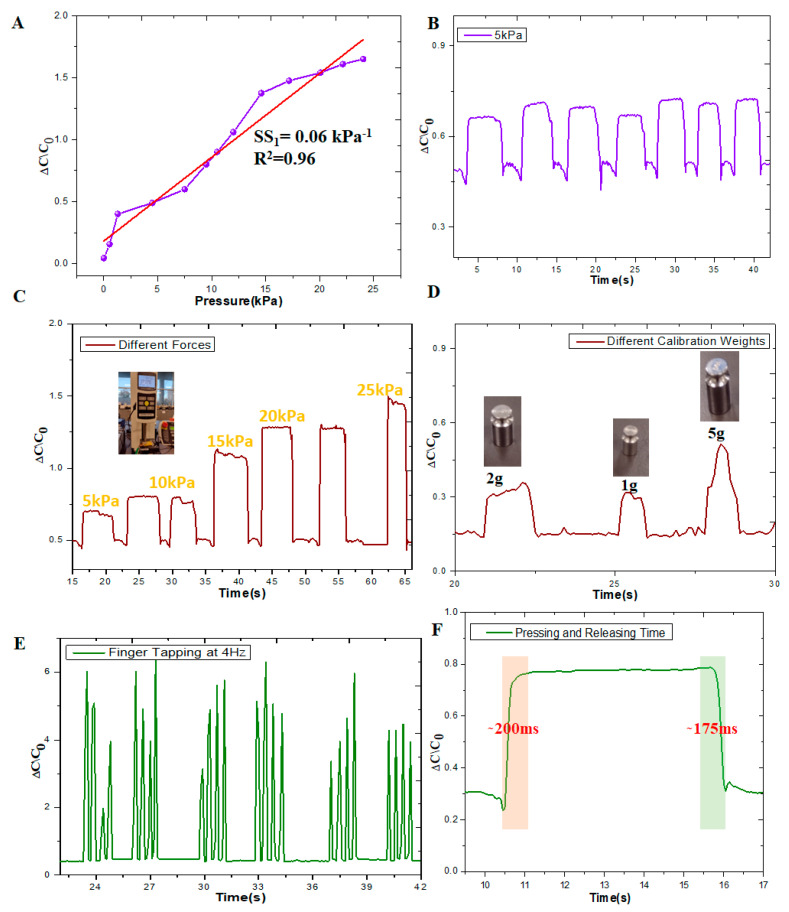
Mechanical characterization under low pressure. (**A**) Measured sensitivity at low-pressure regions of the capacitive sensor fabricated micropatterned PDMS film. (**B**) Measured capacitance under repeated 5 kPa. (**C**) Measured capacitance under repeated varying applied forces. (**D**) Measured capacitance under small loads (1 g, 2 g, and 5 g). (**E**) The sensor’s response to soft finger tapping at approximately 4 Hz frequency. (**F**) Measured response and relaxation time of the sensor.

The fabricated sensor was tested for its pressure response and sensitivities using the measurement system shown in Figure S1. We measured the sensitivity of the fabricated micropatterned PDMS film, as shown in Figure 3A. We obtained the capacitance output under different pressures and calculated the sensitivity and linearity of the fabricated sensor, which are SS_1_ = 0.06 kPa^−1^, R^2^ = 0.96. We manually applied a constant pressure of 5 kPa on the sensor and repeatedly measured the capacitance, as shown in Figure 3B. In repeated loading and unloading scenarios, the capacitance response continued to be constant.

To quantify the sensor error and repeatability, multiple experiments were performed with loading and unloading of the same pressure (5 kPa), as shown in Figure 3B. The results showed that the mean value of ΔC/C_0_ is 0.6940, standard deviation 0.0236, and standard error 0.0016. The mean of the response time was found to be 0.2129 s, the standard deviation of the response time 0.0198, and the standard error of the response time was found 0.0075.

The output capacitance exhibited a consistent shape, although slight variations occurred due to manual pressure application and release. Dynamic force demonstrations were conducted by repetitively touching and withdrawing the force gauge head from a flat surface to apply and remove dynamic pressure, as depicted in Figure 3C. As depicted in Figure 3C, the capacitance increased with higher applied pressure. An essential characteristic of the wearable pressure sensor is its ability to detect low pressures. To assess the performance in the ultra-low-pressure range, sequential loading and unloading of tiny pressures (1, 2, and 5 g) were conducted, and the sensor’s response was analyzed, as shown in Figure 3D. Additionally, the sensor’s capability for detecting delicate pressures was evaluated by tapping and releasing the sensor, as illustrated in Figure 3E. A consistent outcome was obtained when the finger was tapped at a frequency of 4 Hz. The capacitive pressure sensor demonstrated response and relaxation periods of 200 ms and 175 ms, respectively, as presented in Figure 3F. Based on the response of the sensor, we can confirm that the sensor performance is stable under low-pressure conditions.

### 3.4. High-Pressure Performance

**Figure 4:**
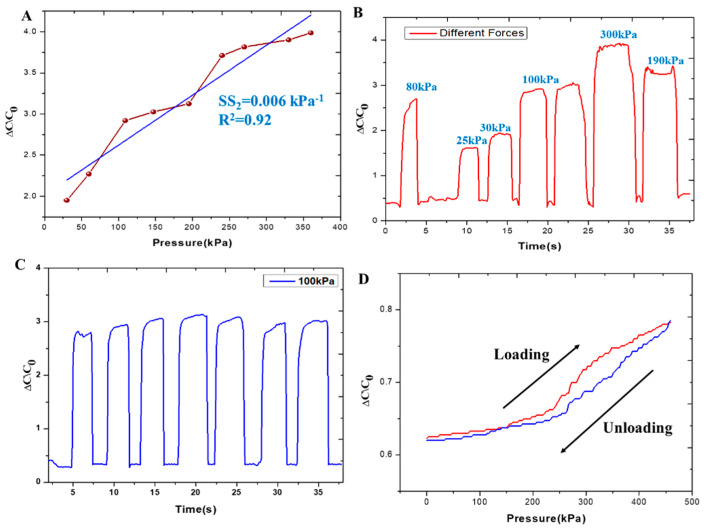
Mechanical characterization under high pressure. (**A**) Measured sensitivity of the capacitive sensor at high pressure regions of the fabricated micropatterned PDMS film. (**B**) Measured capacitance under repeated varying applied forces. (**C**) Measured capacitance under repeated 100 kPa. (**D**) Variation in relative capacitive change while loading and unloading.

The sensor was characterized in the high-pressure range by subjecting it to various forces, as illustrated in Figure 4. The capacitance output was measured at different pressure levels, the linearity of the fabricated sensor in the high-pressure region was calculated with an R^2^ value of 0.92, and the sensitivity was SS_2_ = 0.006 kPa^−1^, as indicated in Figure 4A. Dynamic forces were also applied, as depicted in Figure 4B, revealing an increase in capacitance with increasing pressure. To verify the stability of capacitance under consistent force, a pressure of 100 kPa was applied to the sensor using a force gauge, and the capacitance was repeatedly measured, as shown in Figure 4C [11,12,13]. Figure 4D shows the variation in relative capacitive change for loading and unloading, which indicates low hysteresis. We also verified the capacitance changes under different forces, as shown in Figure S4, compared to Figure 4B. In order to verify the repeatability of the proposed sensor, we used three sensors for obtaining the capacitances under different forces. According to Figure S5, there are slight variations in capacitances. These variations occurred because of the air gap of the capacitive pressure sensor. Since the variations are not large, it shows the repeatability of the proposed sensor.

### 3.5. Gait Signal Acquisition and Analysis

**Figure 5:**
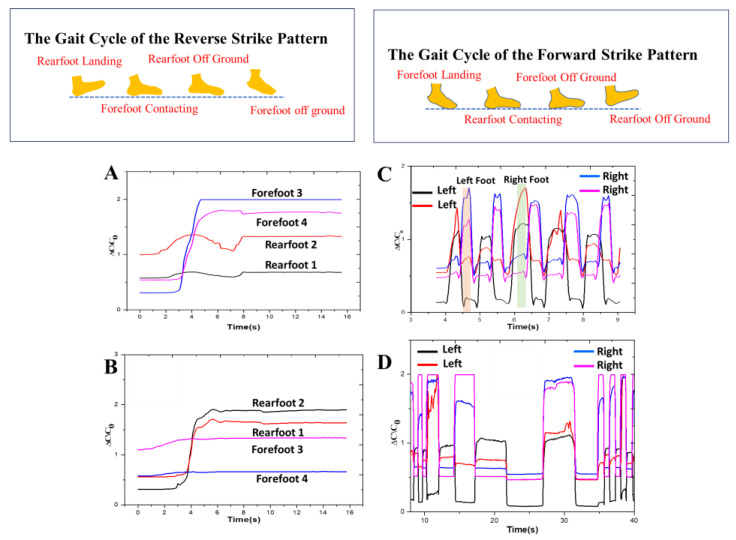
Gait signal acquisition for identification of foot pressure distribution in different dynamic phases. Record of (**A**) the gait cycle of the reverse strike pattern, (**B**) forward strike pattern using the fabricated smart insole system, (**C**) gait signal of the left and right foot under normal walking conditions, and (**D**) putting the left and right foot on different areas under different forces.

The process of tracking gait signals encompasses the collection of diverse parameters associated with an individual’s walking style. The choice of specific signals to observe is contingent upon the goals of the gait analysis and the applications under consideration. In this context, we acquired gait signals during distinct dynamic phases using the proposed wearable device. This device holds potential for applications such as the examination of biomechanics, evaluation of rehabilitation outcomes, and assessment of intervention effects [11,12,13,14,15,16,17,18,19,20,21,22,23,24,25,26,27,28,29,30,31,32,33,34,50]. Technological advancements in sensors, particularly in soft and wireless sensing systems, have significantly increased the ease of monitoring these gait signals across different environments. A recording of four steps utilizing the insole is shown in Figure 5A,B, demonstrating that the distinct gait phases can be identified using the obtained time and capacitance information. The sensors were attached to an insole, demonstrating their capacity to track human gait by analyzing the multiple phases of pressure signals from different foot areas [13].

To capture signals from the foot motion of a human, a basic four-channel capacitance-to-digital converter called FDC1004 was employed and coupled to the pressure sensor, as displayed in Figure 1. The proposed sensor was incorporated into the heel of a commercial insole to demonstrate its application as a smart insole. To perform the experiment measurement, first, we demonstrated and measured the two-gait cycle pattern, which is the forward strike pattern and the reverse strike pattern [14]. Figure 5A shows the obtained capacitance of the reverse strike gait cycle pattern. We placed two sensors at the Rearfoot1 and Rearfoot2 and two sensors at the Forefoot3 and Forefoot4, as shown in Figure S3A. In the case of a reverse strike pattern, first, the subject was asked to land the rear foot and then contact the ground using the forefoot and, finally, lift the rear foot. According to Figure 5A, the obtained capacitance follows the pattern. Sensors Rearfoot1 and Rearfoot2 have higher capacitances than Forefoot3 and Forefoot4 because of the heel pressure. When the forefoot contacted the ground, the rearfoot was lifted, and sensors Rearfoot1 and Rearfoot2 gave higher capacitances than Forefoot3 and Forefoot4 because of the forefoot pressure on the sensor. Figure 5B shows the obtained capacitance of the forward strike gait cycle pattern. It is the opposite case of the reverse strike pattern where, initially, the sensors Forefoot3 and Forefoot4 give higher capacitances compared to Rearfoot1 and Rearfoot2 because of the front foot pressure. Later, when the front foot was lifted, sensors Rearfoot1 and Rearfoot2 gave higher capacitances than Forefoot3 and Forefoot4 because of the rearfoot pressure on the sensor. The obtained capacitance behavior indicates that the sensors are working accurately to detect and monitor the gait cycle pattern. Subsequently, we positioned the sensors on the left and right feet, as shown in Figure S3B, and instructed the subject to imitate a walking pattern. The gait signal was then recorded simultaneously from both feet. The fabricated pressure sensors showed an increase in capacitance when pressed by the foot, owing to the compression of the dielectric layer (micropatterned PDMS), as depicted in Figure 5C [13,34]. Conversely, when the foot was lifted, the capacitance was reduced because of the elastic regaining of the PDMS dielectric. Figure 5D shows the obtained capacitance under different forces on different parts of the left and right foot. We can easily identify the different pressure locations from different areas of the left and right foot.

**Figure 6:**
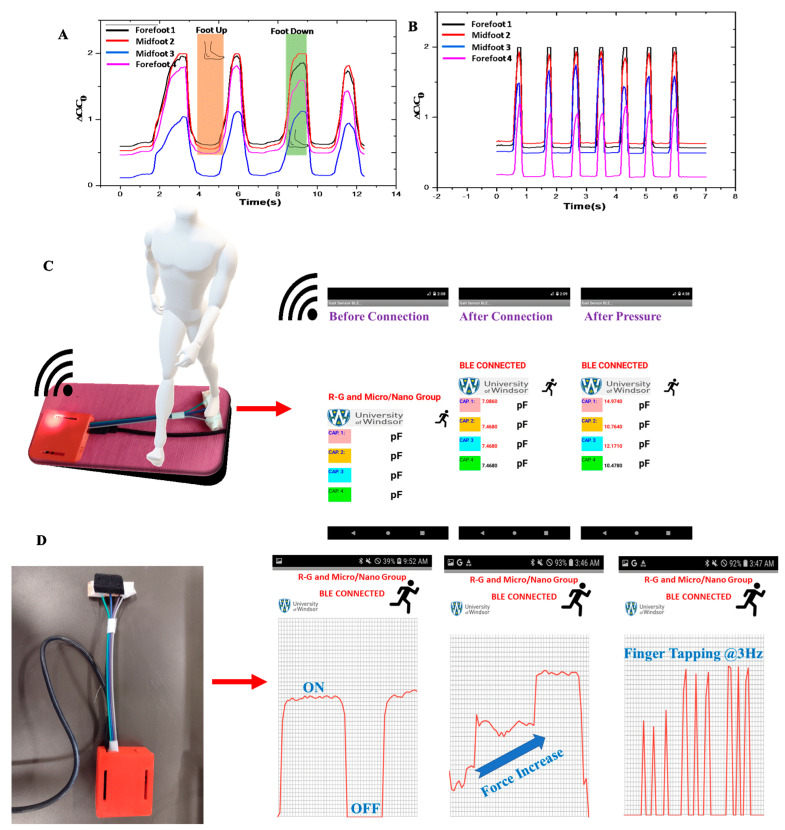
Gait signal acquisition (**A**) limping of the left and right foot, (**B**) foot tapping frequency, (**C**) four sensors data on the Android app, (**D**) single sensor data plotted on the Android app.

We evaluated the limping of the left and right foot under different forces, as demonstrated in Figure S3C. All the sensors responded well according to different dynamic forces. We can identify the location of the force after analyzing the capacitance data, as described in Figure 6A. To check the limping of a foot up and down, we placed the sensors in different locations of a single foot while doing foot up and down motion. Observations revealed that when the foot was elevated, the capacitance decreased as the sensor remained unpressed for specific durations. Conversely, when the foot was lowered, the capacitance increased, with variations depending on the sensor’s location, as depicted in Figure 6A. The recorded capacitance values resulting from foot tapping at a frequency of 7 Hz as shown in Figure 6B. The combination of Figure 6A,B demonstrates that the proposed sensor enables easy differentiation between the gait patterns of the right and left foot.

The developed sensor was integrated into a wearable device, demonstrating its utility in capturing gait signals during various dynamic phases. Recorded signals enabled identification of distinct gait phases, showcasing the sensor’s potential for applications in biomechanics, rehabilitation assessment, and intervention effects monitoring. The sensor’s capability to track pressure signals from different foot areas further enhances its suitability for gait analysis.

### 3.6. Practical Applications and Wireless Monitoring

We made an Android application to monitor the gait signal wirelessly. Figure 6A shows the four capacitances obtained from the single foot using Bluetooth communication. Moreover, we made another Android application to show the pressure ON–OFF result, as shown in Figure 6B. Additionally, to illustrate the screen-printed pressure sensor in dynamic pressure mapping applications, we use finger taps to press the pressure sensor. The signal from the pressure sensor changes immediately, as shown in Figure 6C,D. This study illustrated practical applications of the sensor, including incorporation into smart insoles for gait analysis. The sensor accurately captured gait cycle patterns, facilitating differentiation between forward and reverse strike patterns. Furthermore, wireless monitoring capabilities were demonstrated through Android applications, enabling real-time gait signal tracking. Dynamic pressure mapping applications were also showcased, highlighting the sensor’s versatility in various monitoring scenarios.

In summary, the developed pressure sensor exhibits promising performance characteristics, making it a valuable tool for precise gait analysis and monitoring in both clinical and research settings. Its soft, flexible nature and wireless capabilities enhance its usability across diverse applications, paving the way for advancements in biomechanics and rehabilitation sciences. Table 1 shows the performance summary of the proposed device. The performance comparison of the proposed sensor with other reported capacitive pressure sensors for gait monitoring is shown in Table S1 (Supporting File).

## 4. Conclusions

In this work, we successfully designed and fabricated a flexible capacitive pressure sensor that utilized micropatterned PDMS dielectric material to analyze human gait. The new integrated sensor design was found to be lightweight, and the robust smart insole developed was used for real-time gait pattern analysis. The new device underwent real-time gait response testing and demonstrated its efficiency in sensing different gait patterns in real-time. A total of four sensors in an integrated assembly were developed for a soft shoe sole, and the device was effectively integrated into a commercially available shoe pad to analyze gait pressure signals. The thickness of the pressure sensor was decreased by utilizing thin and flexible polymers (PDMS) and a thin patterned electrode. The design of the sensor was fine-tuned using microfabrication to make it thinner and flexible while still maintaining sensitivity and accuracy. Additionally, the pressure sensor was combined with active electronic components, such as a capacitance-to-voltage digital converter and wireless transmitters, to increase the data connectivity, and decrease its overall size and thickness while preserving its functionality. This can be beneficial in instances where there is limited space in the shoe, or the sensor needs to be incorporated into a larger system. The findings indicate that the flexible pressure sensor created in this study is a valuable instrument for analyzing gait signals and differentiating various walking patterns. The simple approach employed to produce sensitive and lightweight sensors demonstrated in this research would be well-suited for detecting human motion and further enabling personalized healthcare applications. The insole has been evaluated in both static and dynamic situations, allowing in the future to track and potentially modify a patient’s problematic posture and walking patterns. Physiotherapists, for example, such information may improve patient recovery by monitoring and fine-tuning the real stress on the limb, while sports professionals may utilize the device to follow an athlete’s training and improve performance.

## Figures and Tables

**Table 1:** Performance summary of the proposed gait monitoring device.

Parameters	Measured Value/Properties/Justifications
Pressure Range	From 0 to 1000 kPa covers the typical pressures exerted by the foot during activities like walking, running, and standing. We evaluated 0 to 300 kPa.
Sensitivity	A range of 0.001–10 kPa^−1^ pressure change is what the sensor should detect. The proposed flexible capacitive pressure sensor exhibits high sensitivity and linearity under low pressure (SS_1_ = 0.06 kPa^−1^, R^2^ = 0.96) and high pressure (SS_2_ = 0.006 kPa^−1^, R^2^ = 0.92)
Accuracy	The desired level of accuracy for pressure measurements is 90% to 95%. To quantify the sensor error and repeatability, multiple experiments were performed with loading and unloading of the same pressure (5 kPa), as shown in Figure 3B. Results showed that the mean value of ΔC/C_0_ is 0.6940, standard deviation is 0.0236, and standard error is 0.0016.
Precision	Accurate measuring is needed to catch small changes in how people walk, so we can study them closely and see how they improve over time. For that reason, we measured the tiny pressure (1 g weight).
Reliability	The proposed system gives the same results each time we measure.
Linearity	The sensor’s response is linear across the pressure range. The flexible capacitive pressure sensor exhibits high sensitivity and linearity under low pressure (SS_1_ = 0.06 kPa^−1^, R^2^ = 0.96) and high pressure (SS_2_ = 0.006 kPa^−1^, R^2^ = 0.92).
Real-Time Monitoring	We made real-time monitoring capability that allows us to see the changes in capacitances with load in real-time.
User-Friendly Interface	We used a simple microcontroller-based user interface and software for the data collection and analysis.
Durability	We used PDMS as the substrate which is durable construction and robust materials ensure the longevity and reliability of the gait sensing system.
Portability	To enhance portability, we designed the data acquisition device to be rechargeable and portable, utilizing lithium polymer (Li-Po) batteries that included built-in protection circuitry. App-based data connectivity.
Hysteresis	Minimize the difference in sensor output for the same pressure, depending on whether the pressure is increasing or decreasing. Figure 4D shows the variation in relative capacitive change for loading and unloading which indicates low hysteresis.
Response Time	The capacitive pressure sensor demonstrated response and relaxation periods of 200 ms and 175 ms, respectively, as presented in Figure 3F.
Stability	The sensor’s performance remains consistent over time, according to Figure 5C,D.
Size and Form Factor	The thickness of the dielectric layers is approx. 3 mm, and the size of the sensor is approx. 2.5 cm × 4 cm. In addition, the outer diameter of the sensor and the diameter of the detection area (i.e., the electrodes) were approximately 2.5 mm and 7 mm, respectively.

## Data Availability

The data used in this study is available from the corresponding authors with a reasonable request.

## Supplementary Materials

The following supporting information can be downloaded at: https://www.mdpi.com/article/10.3390/s24092944/s1, Figure S1: Measurement setup. (A) Setup for the measurement of the sensor response. (B) Photograph of gait signal acquisition using a flexible capacitive pressure sensor. (C) Image of the flexible electrode surface. (D) SEM image of the micropatterned PDMS. The white scale bar represents 300 µm. Figure S2: (A) Experimental image of the electrical characterization, (B) Resistance changes of electrodes when bent on PDMS. Figure S3. Placement of sensors in different locations. (A) two forefoot and two rearfoot, (B) left and right side (forefoot and rearfoot), (C) one forefoot, two midfoot, and one rearfoot. Figure S3: Placement of sensors in different locations. (A) two forefoot and two rearfoot, (B) left and right side (forefoot and rearfoot), (C) one forefoot, two midfoot, and one rearfoot. Figure S4: Capacitance changes under different forces. Figure S5: Capacitance changes under different forces for three sensors; Table S1. Performance comparison of capacitive pressure sensor for gait monitoring application.
