# Supplementary material for: Gait Pattern Analysis: Integration of a Highly Sensitive Flexible Pressure Sensor on a Wireless Instrumented Insole
Source: Sensors (Basel). 2024 May 6;24(9):2944. doi: 10.3390/s24092944 (PMC11086061; doi:10.3390/s24092944)
Supplement: Supplementary file 1 [file sensors-24-02944-s001.zip › sensors-2895255-supplementary.pdf]

Gait Pattern Analysis: Integration of a Highly Sensitive Flexible Pressure Sensor on Wireless Instrumented Insole

Partha Sarati Das[1], Daniella Skaf[2], Lina Rose[1], Fatemeh Motaghedi,[2] Tricia Breen Carmichael[2], Simon Rondeau-Gagné[2]*, Mohammed Jalal Ahamed[1]*

[1]Mechanical, Automotive & Materials Engineering, University of Windsor, Ontario, Canada.

[2]Department of Chemistry and Biochemistry, University of Windsor, Ontario, Canada.

*Corresponding author: Prof. Dr. Mohammed Jalal Ahamed (email: jahamed@uwindsor.ca).

Supporting Information

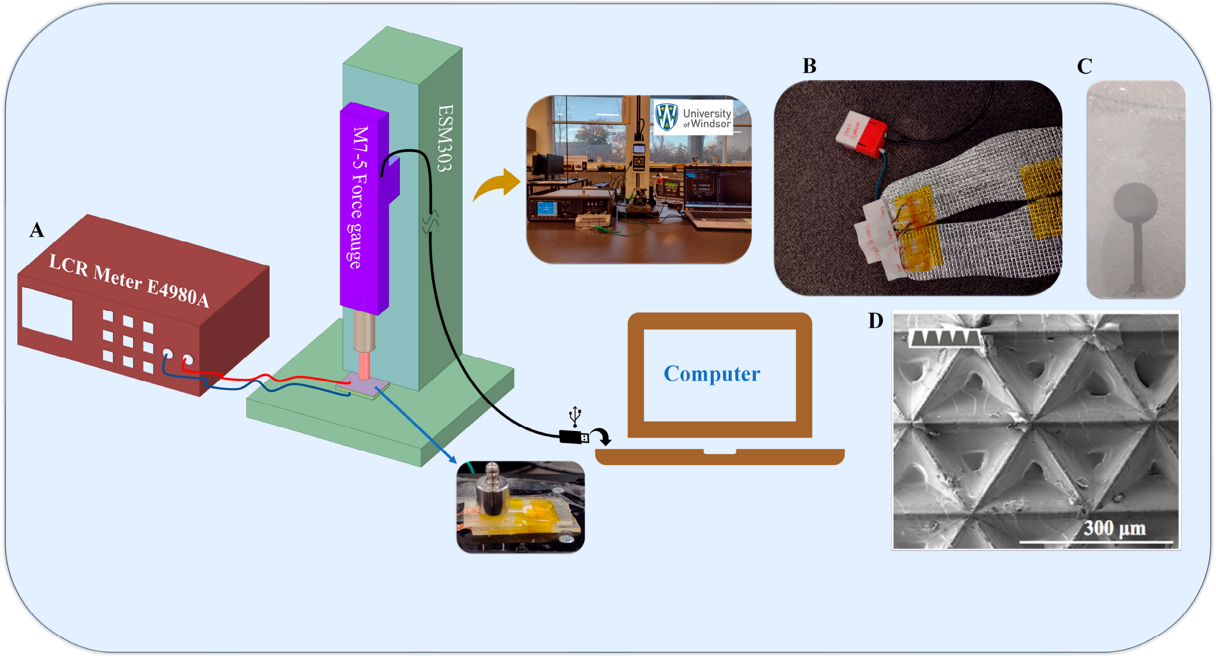

Figure S1. Measurement setup. (A) Setup for the measurement of the sensor response. (B) Photograph of gait signal acquisition using a flexible capacitive pressure sensor. (C) Image of the flexible electrode surface. (D) SEM image of the micropatterned PDMS. The white scale bar represents 300 μm.

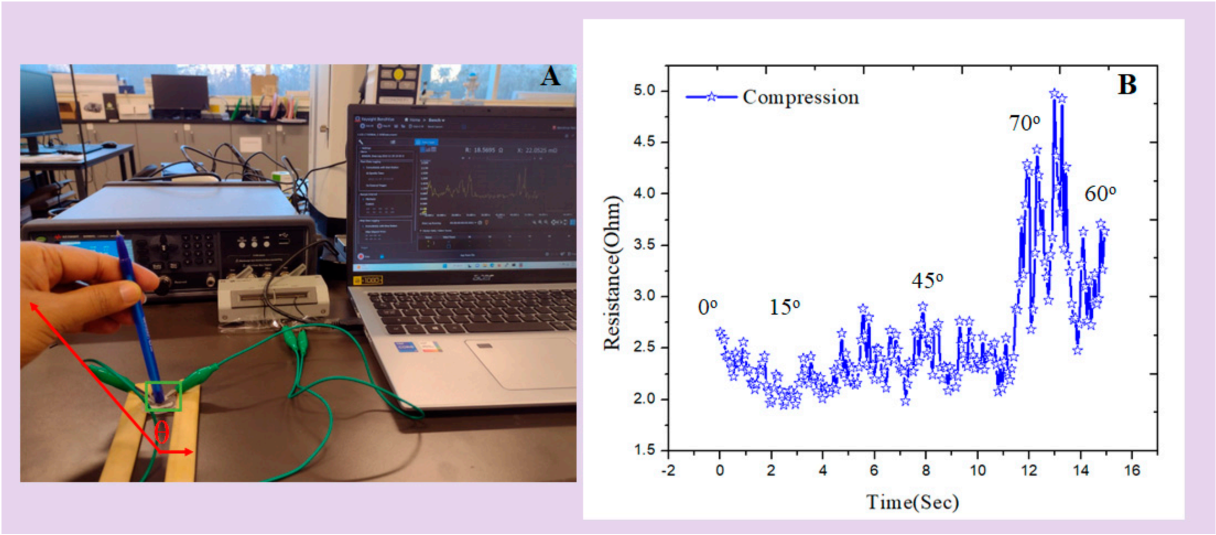

Figure S2. (A) Experimental image of the electrical characterization, (B) Resistance changes of electrodes when bent on PDMS

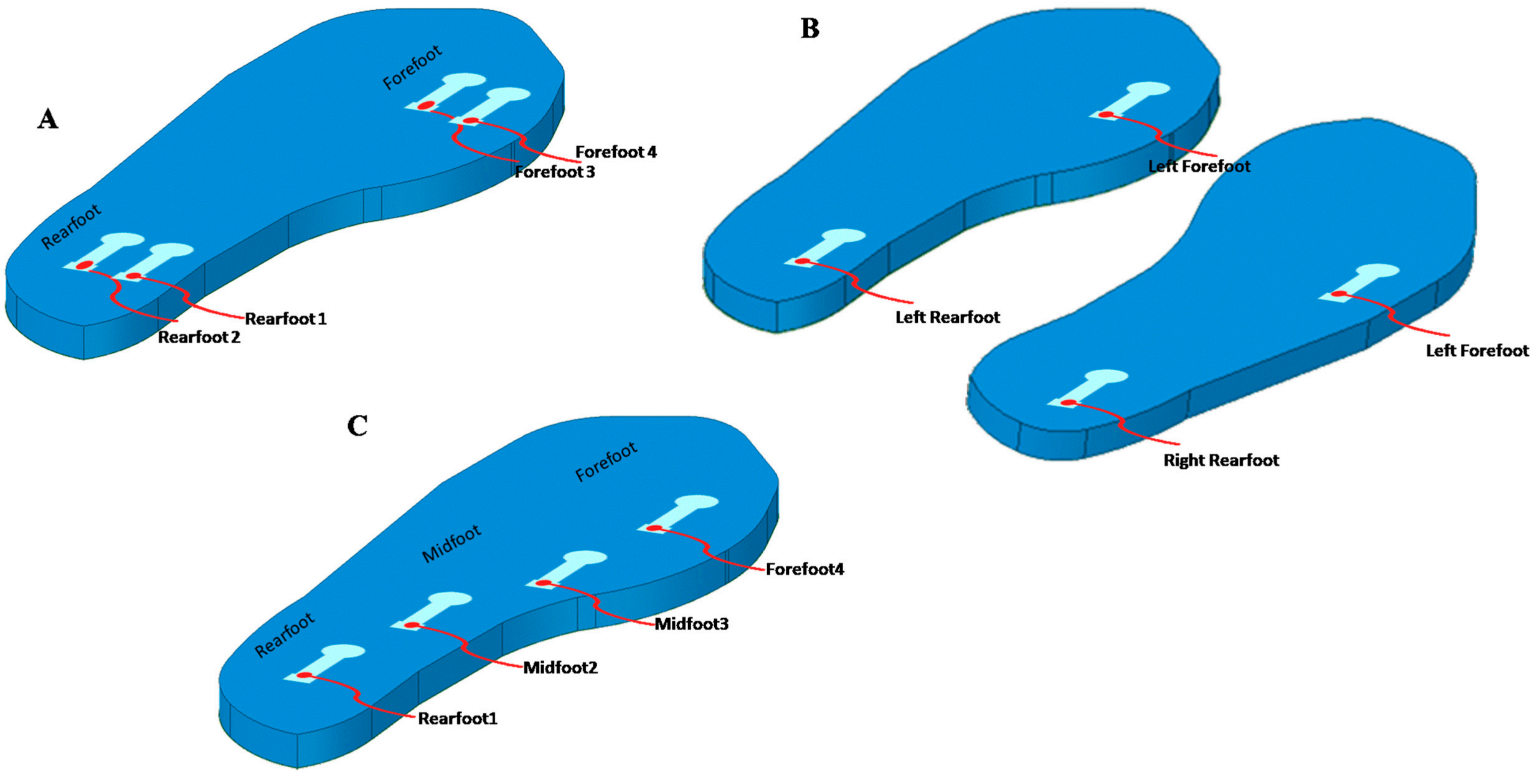

Figure S3. Placement of sensors in different locations. (A) two forefoot and two rearfoot, (B) left and right side(forefoot and rearfoot), (C) one forefoot, two midfoot, and one rearfoot.

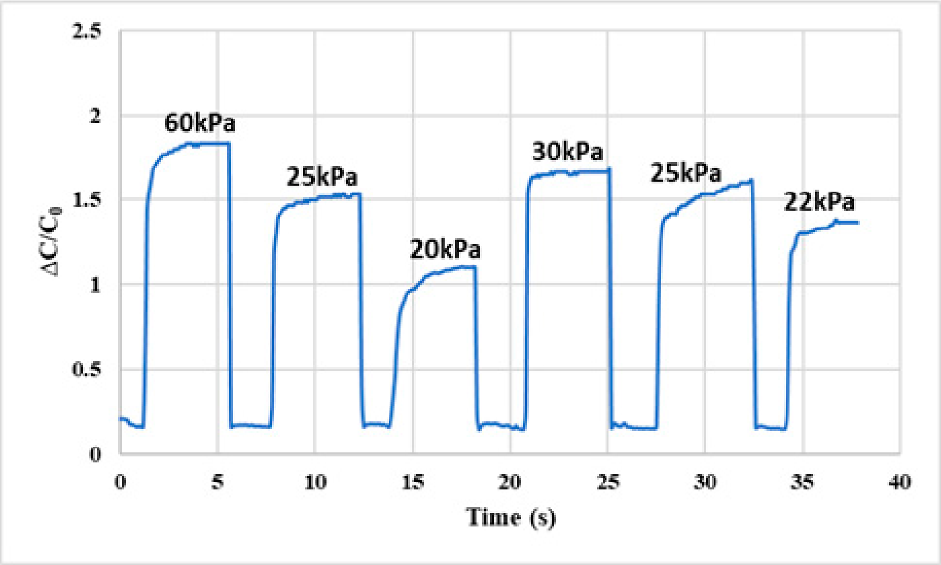

Figure S4. Capacitance changes under different forces.

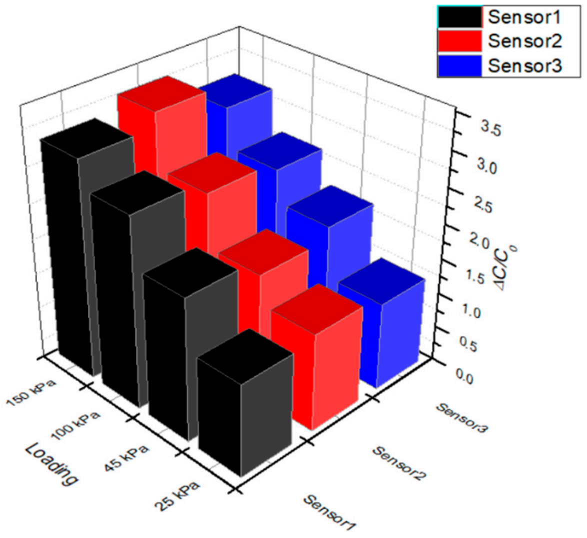

Figure S5. Capacitance changes under different forces for three sensors.

Table S1. Performance comparison of capacitive pressure sensor for gait monitoring application

Year	Sensors used	Sensitivity/Linearity	Materials	No. of Sensors	Location of Sensor	Gait Parameters	Sensor Parameters
2020 [11]	Capacitive pressure sensor	12×10^{-3} kPa^{-1}	Ecoflex, Conductive fabric	24	5 heel, 7 middle, and 12 in forefoot	Standing postures, yoga asana, walking straight, turning around, falling, and going upstairs	Pore size, sensitivity, linearity, stability
2022 [12]	Capacitive pressure sensor	Different under different pressure	3D printed, paper based AgNW electrode	3	Walking pressure at heel and tiptoe	Walking pattern	Sensitivity, detection range, stability
2022 [13]	Capacitive pressure sensor	1.19±0.03MPa^{-1}	Filaflex 82A	16	Calcaneus, plantar arch, metatarsals, and phalanges	Toe off, stance, heel strike, foot flat, midstance, and heel off	Dynamic response, sensitivity, linearity, pressure detection range, stability, durability, hysteresis, response time
2022 [14]	Capacitive pressure sensor	1.314MPa^{-1}	TPU, EITPU, Eco flex	4	1 Heel, 1 right, 1 left, and 1 toe	Steps of walking	Linearity, efficiency, durability, sensitivity
2022 [16]	Capacitive pressure sensor	0.953	TPU, PDMS, MWCNT, AgNP	5	Different locations	Sitting, sit-to-stand transition, standing, walking, and turning	Sensitivity, hysteresis, and durability
2020 [34]	Capacitive pressure sensor		PCB based	4	Heel	Heel strike, toe off	Specificity and sensibility
This Work	Capacitive pressure sensor	Linearity, R^2=0.96, Slope=0.14	PDMS, screen-printed silver (Ag) paste	4	1 rearfoot, 2 midfoot, 1 forefoot	Reverse strike pattern, forward strike pattern, walking	Detection range, response time, relaxation time, Linearity, durability
